# Supplementary figures and images for: Role of miR-10b-5p in the prognosis of breast cancer
Source: PeerJ. 2019 Sep 20;7:e7728. doi: 10.7717/peerj.7728 (PMC6756141; doi:10.7717/peerj.7728)

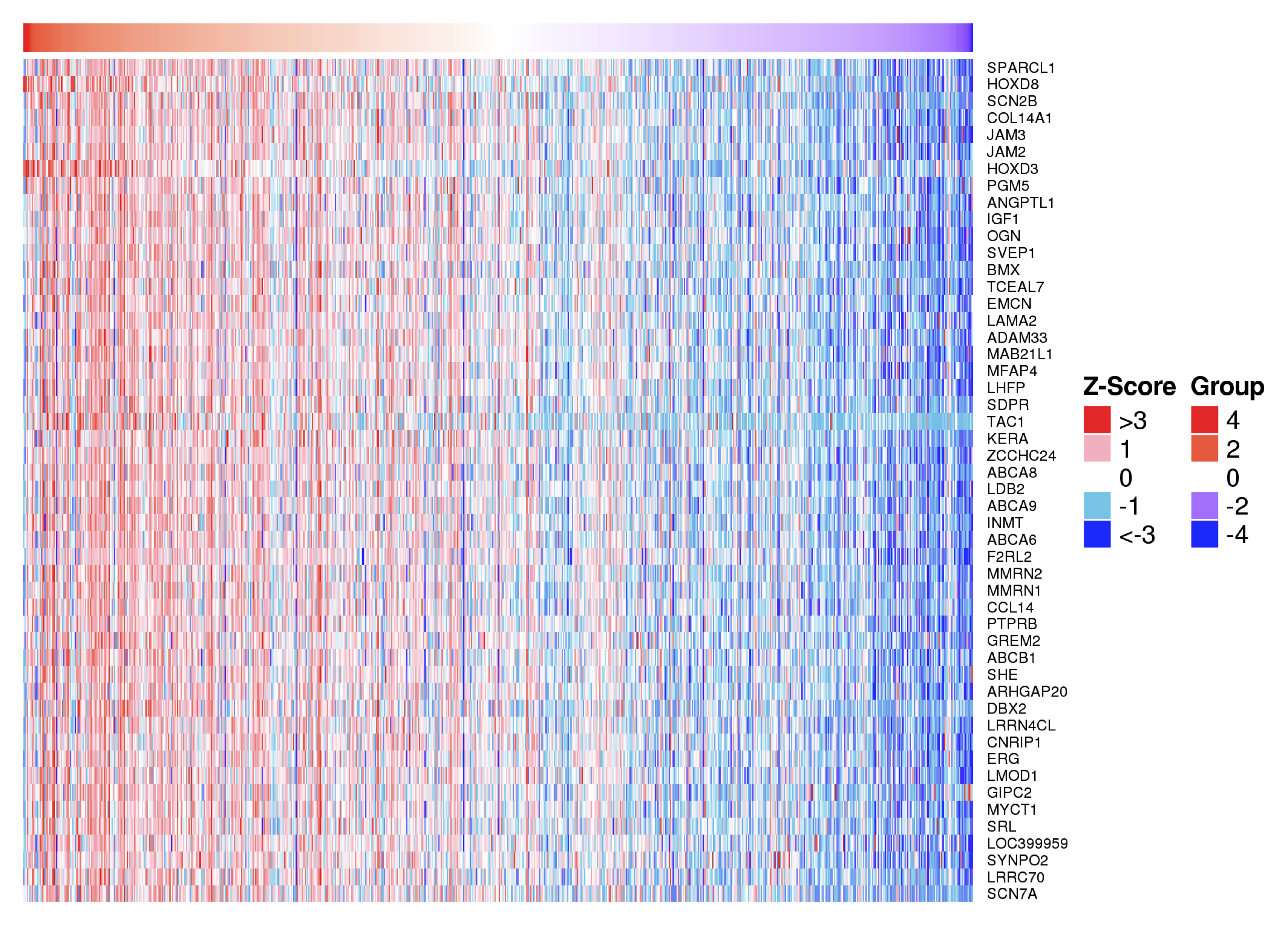

Supplement: Figure S1 — A total of 50 significantly positively correlated genes were acquired from LinkedOmics. [file peerj-07-7728-s001.png]

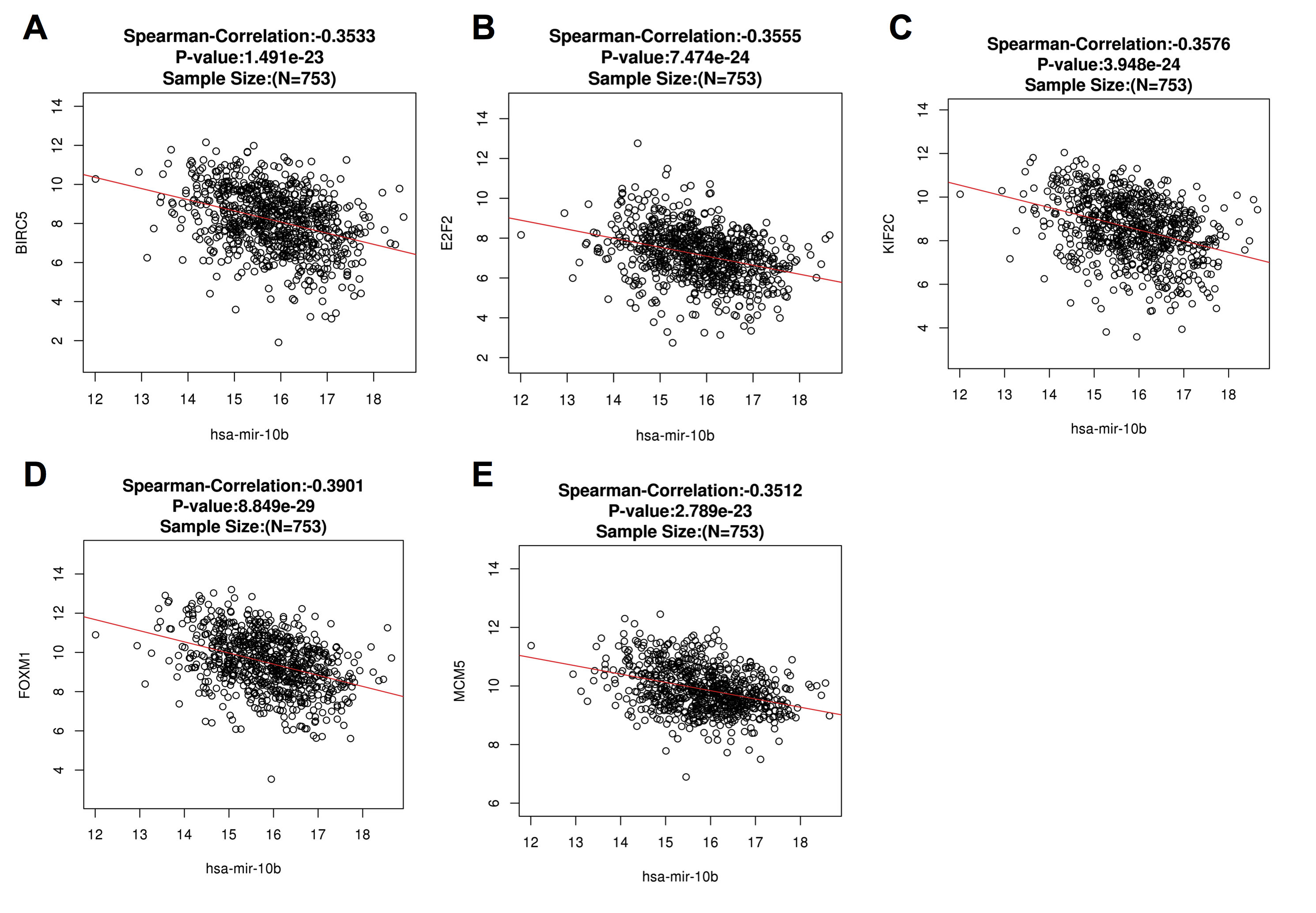

Supplement: Figure S2 — Scatter plots showing the inverse relation between miR-10b-5p and its target genes in breast cancer. [file peerj-07-7728-s002.png]
