# Supplementary material for: Role of miR-10b-5p in the prognosis of breast cancer
Source: PeerJ. 2019 Sep 20;7:e7728. doi: 10.7717/peerj.7728 (PMC6756141; doi:10.7717/peerj.7728)
Supplement: Table S1 [file peerj-07-7728-s003.docx]

| Query | Statistic | P-value | FDR (BH) | Statistical method |
| --- | --- | --- | --- | --- |
| Pathologic_stage | 4.11 | 0.250 | 0.311 | Kruskal-Wallis Test |
| Pathology_T_stage | 4.05 | 0.257 | 0.311 | Kruskal-Wallis Test |
| Pathology_N_stage | 6.11 | 0.107 | 0.181 | Kruskal-Wallis Test |
| Pathology_M_stage | 0.021 | 0.586 | 0.622 | Wilcox Test |
| PR. Status | 0.007 | 0.507 | 0.575 | Wilcox Test |
| HER2. Status | 0.012 | 0.201 | 0.285 | Wilcox Test |

**Table S1 Associations between has-miR-10b and clinical characteristics analyzed using LinkedOmics.**

P-value: P-value obtained from statistical method.

FDR (BH): FDR is calculated by BH (Benjamini-Hochberg method).
