# Supplementary material for: Role of miR-10b-5p in the prognosis of breast cancer
Source: PeerJ. 2019 Sep 20;7:e7728. doi: 10.7717/peerj.7728 (PMC6756141; doi:10.7717/peerj.7728)
Supplement: Table S2 [file peerj-07-7728-s004.docx]

**Table S2 Interactions between miR-10b-5p and target mRNAs.**

| Gene | Class | Alignment |
| --- | --- | --- |
| BIRC5 | 6mer | Target: 5' guggcugcaccacuucCAGGGUu 3'                              miRNA: 3' guguuuaagccaagauGUCCCAu 5' |
| E2F2 | 6mer | Target: 5' agacgagggauuauuuCAGGGUg 3'                           miRNA: 3' guguuuaagccaagauGUCCCAu 5' |
| KIF2C | 6mer | Target: 5' guaccuggUGGGUCUAGGCAGGGUc 3'                  ： ： miRNA: 3' guguuuaaGCCAAGA----UGUCCCAu 5' |
| FOXM1 | 6mer | Target: 5' ccuucccugaucUUUGCAGGGUg 3'                   ： ：  miRNA: 3' guguuuaagccaAGAUGUCCCAu 5' |
| MCM5 | 7mer-m8 | Target: 5' gaCAAGGUCGUCCCUGGGAACAGGGUu 3'               ：  miRNA: 3' guGUUUAAGCCAAGA---------UGUCCCAu 5' |
